# An Altered gp100 Peptide Ligand with Decreased Binding by TCR and CD8α Dissects T Cell Cytotoxicity from Production of Cytokines and Activation of NFAT

**DOI:** 10.3389/fimmu.2013.00270

**Published:** 2013-09-04

**Authors:** Niels Schaft, Miriam Coccoris, Joost Drexhage, Christiaan Knoop, I. Jolanda M. de Vries, Gosse J. Adema, Reno Debets

**Affiliations:** ^1^Laboratory of Experimental Tumor Immunology, Department Medical Oncology, Erasmus MC Cancer Institute, Rotterdam, Netherlands; ^2^Department Tumor Immunology, Nijmegen Center for Molecular Life Sciences, Radboud University Nijmegen Medical Center, Nijmegen, Netherlands

**Keywords:** activation of nuclear factor of activated T cells, altered peptide ligands, cytokine production, cytotoxicity, human T lymphocytes, T cell receptor

## Abstract

Altered peptide ligands (APLs) provide useful tools to study T cell activation and potentially direct immune responses to improve treatment of cancer patients. To better understand and exploit APLs, we studied the relationship between APLs and T cell function in more detail. Here, we tested a broad panel of gp100_280–288_ APLs with respect to T cell cytotoxicity, production of cytokines, and activation of Nuclear Factor of Activated T cells (NFAT) by human T cells gene-engineered with a gp100-HLA-A2-specific TCRαβ. We demonstrated that gp100-specific cytotoxicity, production of cytokines, and activation of NFAT were not affected by APLs with single amino acid substitutions, except for an APL with an amino acid substitution at position 3 (APL A3), which did not elicit any T cell response. A gp100 peptide with a double amino acid mutation (APL S4S6) elicited T cell cytotoxicity and production of IFNγ, and to a lesser extent TNFα, IL-4, and IL-5, but not production of IL-2 and IL-10, or activation of NFAT. Notably, T cell receptor (TCR)-mediated functions showed decreases in sensitivities for S4S6 versus gp100 wild-type (wt) peptide, which were minor for cytotoxicity but at least a 1000-fold more prominent for the production of cytokines. TCR-engineered T cells did not bind A3-HLA-A2, but did bind S4S6-HLA-A2 although to a lowered extent compared to wt peptide-HLA-A2. Moreover, S4S6-induced T cell function demonstrated an enhanced dependency on CD8α. Taken together, most gp100 APLs functioned as agonists, but A3 and S4S6 peptides acted as a null ligand and partial agonist, respectively. Our results further suggest that TCR-mediated cytotoxicity can be dissected from production of cytokines and activation of NFAT, and that the agonist potential of peptide mutants relates to the extent of binding by TCR and CD8α. These findings may facilitate the design of APLs to advance the study of T cell activation and their use for therapeutic applications.

## Introduction

T lymphocytes are potent mediators of anti-tumor immune responses. In fact, T cell receptor (TCR) genes derived from anti-tumor T lymphocytes have been successfully used to redirect other, non-tumor-specific T lymphocytes to tumor cells, and have shown promising clinical activities in the treatment of tumor-bearing patients (1, 2). Adoptive T cell therapy to tumors is based on the ability of TCRs to selectively recognize antigens, i.e., peptides that are presented by Major Histocompatibility Complex (MHC) molecules. The clinical use of TCR-engineered T lymphocytes directed against the human leukocyte antigen (HLA)-A2-restricted antigens MART-1, gp100, or NY-ESO-1 resulted in objective responses in patients with metastatic melanoma up to 45% (3, 4). Importantly, the avidity and antigen reactivity of parental T cell clones, used as a source for TCR genes, are preserved by TCR gene transfer (5–7). Moreover, cytotoxic responses of TCR-engineered T cells toward a panel of gp100 peptide mutants are identical to those of parental CTL clones (7).

Studies with mutated peptides, so called altered peptide ligands (APLs), have eloquently demonstrated that T cell recognition of antigen is flexible and that binding of different APLs can result in distinct and selective T cell signaling and functions (8, 9). APLs can be classified depending on the T cell responses they elicit; e.g., agonists induce the full range of T cell activation such as proliferation, cytokine secretion, and cytotoxic killing; partial agonists sub-optimally activate T cells and cause a selective pattern of effector functions; null agonists do not activate T cells; whereas antagonists specifically inhibit T cell activation induced by the wild-type (wt) peptide [reviewed in (10, 11)]. Interestingly, melanoma cells can process and present antagonistic APLs themselves, thereby potentially providing cues that prevent maximal intra-tumoral T cell activation and facilitate immune evasion (12). Immune suppression mediated by antagonistic peptide variants can be reversed by APLs with highly agonist properties that are able to sensitize T cells and yield resistance against effects of inhibitory APLs (12, 13). Importantly, APLs have already been used in immunotherapeutic strategies with the intent to more effectively skew immune responses against autoimmune diseases, infectious diseases, and cancer [reviewed in (10)].

Numerous APLs have been designed for cancer epitopes and include, amongst others, MUC1-HLA-A2 (14), HER1-HLA-A2 (15), HER2-HLA-A2 (16), HER2-HLA-A24 (17), MelanA-HLA-A2 (18), gp100-HLA-A2 [epitopes 154, 209, and 280 (19)], TRP2-HLA-A2 (20), PSA-HLA-A2 (21), and NY-ESO1-HLA-A2 (22). Such APLs have principally been designed to improve the binding affinity of peptide to the MHC molecule, allowing induction of improved T cell responses against wt epitope. For example, MelanA-HLA-A2-specific T cell responses have rapidly and reproducibly been induced with the highly immunogenic APL with a Leucine at anchor position 2 (L2) (18, 23). However, enhanced immunogenicity of APLs may not necessarily be accompanied by the induction of a curative T cell response specific for the native epitope in patients with cancer. In fact, the modified MelanA epitope may alter TCR binding and prime T cells with different TCRs compared to the wt peptide (24). Indeed in patients with melanoma, T cells elicited by APL L2 demonstrated higher frequencies but weaker functional T cell avidity toward the native epitope (25). This is not necessarily a general finding as gp100 APLs (gp100_154–162_ A8 and gp100_280–288_ V9) were clinically equally effective when compared to wt peptides when used in combination with a DC vaccine (26). Collectively, however, these studies challenge the value and clinical applicability of APLs. Further and detailed studies into APLs and their effects on various T cell parameters are needed to gain a better understanding of the perimeters of T cell specificity and sensitivity. In addition, a correct definition of agonist and potential antagonist properties of APLs will allow successful translation of selected APLs to clinical settings. It is noteworthy that besides the setting of vaccination, where the frequency of the relevant TCR may be insufficient, the clinical potential of APLs may be extended to the setting of adoptive T cell therapy, which ensures a high frequency of the expected TCR in patients.

Here, we have used a panel of gp100_280–288_ APLs and explored APL characteristics in relation to T cell recognition and different T cell responses. To this end, we have transferred a defined TCR, i.e., a gp100-HLA-A2-specific TCR, into human T cells, and tested the effect of individual and double amino acid substitutions of the wt gp100 peptide on T cell responses. Analyses of gp100 APLs revealed that all single amino acid mutants retain their agonistic properties, except for the A3 mutant and double amino acid S4S6 mutant that acted as a null ligand and partial agonist, respectively. Findings showed that TCR-mediated cytotoxicity can be dissected from production of cytokines and activation of nuclear factor of activated T cells (NFAT), and suggest that the agonist potential of APLs relates to the extent a peptide mutant is bound by TCR and CD8α.

## Materials and Methods

### Cells and reagents

Peripheral blood lymphocytes (PBL) from healthy donors were isolated by centrifugation through Ficoll-Isopaque (density = 1.077 g/cm^3^; Pharmacia Biotech, Uppsala, Sweden). Obtaining and handling of human samples, such as PBL, were according to national and institutional guidelines and approved by the Erasmus MC Cancer Institute’s ethical committee. Primary human T lymphocytes were cultured as described elsewhere (7). The TAP-deficient TxB cell hybrid and HLA-A2-positive T2, and the gp100-positive, HLA-A2-positive melanoma cell line FM3 were maintained in DMEM (Gibco BRL, Paisley, Scotland, UK) supplemented with 10% Bovine Calf Serum (BCS: Hyclone, Logan, UT, USA) and the antibiotics streptomycin (100 μg/ml) and penicillin (100 U/ml). The HLA-A2-positive melanoma cell lines BLM and BLMgp100 (the latter transfected with human gp100-encoding cDNA) were cultured as described previously (27, 28). The Jurkat T cell clone E6.1 was expanded in RPMI 1640 medium supplemented with l-glutamine, 10% BCS, and antibiotics.

### Peptides and peptide-MHC multimers

Peptides used in this study were: the gp100_280–288_ wt peptide YLEPGPVTA, the gp100 APLs A1–A8, G9, and S4S6, indicating an Alanine, Glycine, or Serine substitution at the indicated amino acid position of the wt peptide, and an irrelevant HLA-A2-binding EBV BMLF-1 wt peptide (GLCTLVAML). Peptide preparations were synthesized as described earlier (7) and found to be>90% pure as analyzed by analytical HPLC. MHC class I binding of peptides was analyzed via stabilization of HLA-A2 on T2 cells, as described previously (29, 30). The gp100 wt peptide, the gp100 APLs A3 and S4S6, and the BMFL-1 wt peptide were used to generate peptide-HLA-A2 monomers (Sanquin Blood Supply Foundation, Amsterdam, Netherlands). Multimers of peptide and HLA-A2 were freshly prepared by incubating streptavidin^PE^ and the corresponding soluble monomers at a 1:4 molar ratio for 1 h at 4°C as described elsewhere (31).

### Cloning and transfer of TCR genes

Genes encoding gp100_280–288_-HLA-A2-specific TCRαβ were PCR-amplified from CTL clone 296 (CTL-296) and cloned into the retroviral vector bullet, as described previously (7). Primary human T lymphocytes of healthy donors, pre-activated with anti-CD3 mAbs were transduced with TCR-positive retroviruses produced by the packaging cell line Phoenix-Amp (32, 33). A retroviral vector encoding human CD8α (34) was used to transduce Jurkat T cells prior to transfer of TCR genes (35). Transduction with Mock genes served as a negative control.

### Flow cytometry and cell-sorting

T cells were analyzed for TCR transgene expression by flow cytometry using either PE-conjugated anti-TCRVβ27 mAb (Beckman-Coulter, Marseille, France) (nomenclature of TCRV regions according to http://imgt.org) or PE-conjugated gp100 wt peptide-HLA-A2 complexes (ProImmune Ltd., Oxford, UK), as described previously (7, 35). In short, 0.1–0.5 × 10^6^ T cells were incubated with mAb on ice for 30 min or peptide-MHC complexes (see Peptides and Peptide-MHC Multimers) at room temperature for 1 h, washed, fixed (1% PFA). T cells were gated according to forward and sideward scatter properties using a FACSCalibur (Becton Dickinson, Alphen a/d Rijn, Netherlands) equipped with CellQuest software (BD Biosciences). Binding of peptide-MHC was analyzed within viable gate of TCR-engineered T cells with marker set at<1% positive binding for non-stained TCR-engineered T cells. TCR-engineered T cells were MACS-enriched using gp100 wt peptide-HLA-A2 multimers and anti-PE MACS MicroBeads according to the manufacturer’s instructions (Miltenyi Biotec, Bergisch Gladbach, Germany).

### Cytotoxicity assay

Cytotoxic activity of T cells was assayed in standard 6 h ^51^Cr-release assays using the following target cells: T2 cells pulsed with 1 μM or titrated amounts of either gp100 wt peptide, gp100 APLs, BMLF-1 wt peptide, or the melanoma cell lines BLM, BLMgp100, or FM3. Antigen-specificity was confirmed by addition of anti-TCRVβ27 mAb or mouse immunoglobulin (mIg; Jackson ImmunoResearch Laboratories, West Grove, PA, USA) (both at 1 μg/ml final), and the contribution of CD8 interactions was studied by addition of anti-CD8α mAb (clone 4H8, 10 μg/ml final, Sanquin Blood Supply Foundation) to T cells 30 min before cultivation with target cells. To inhibit NK cell activity during the cytotoxicity assay, an excess of non-labeled K562 cells was added to target cells prior to assay. The percentage of specific ^51^Cr-release was determined as follows: [(test counts − spontaneous counts)/(maximum counts − spontaneous counts)] × 100% (36).

### Cytokine measurements

To quantify the production of cytokines, 6 × 10^4^ T cells were cultured in the presence of 2 × 10^4^ T2 cells pulsed with titrated amounts of peptide for 18 h. Supernatants were harvested, and levels of IL-2, IL-4, IL-5, IL-10, IFNγ, and TNFα were determined via Cytokine Bead Array (CBA kit; BD Biosciences), according to the manufacturer’s instructions. In some experiments, CBA data were supplemented with ELISAs (Sanquin Blood Supply Foundation), which were performed according to the manufacturer’s instructions. As a positive control, T cells were stimulated with PMA and PHA.

### NFAT reporter gene assay

**Figure 1:**
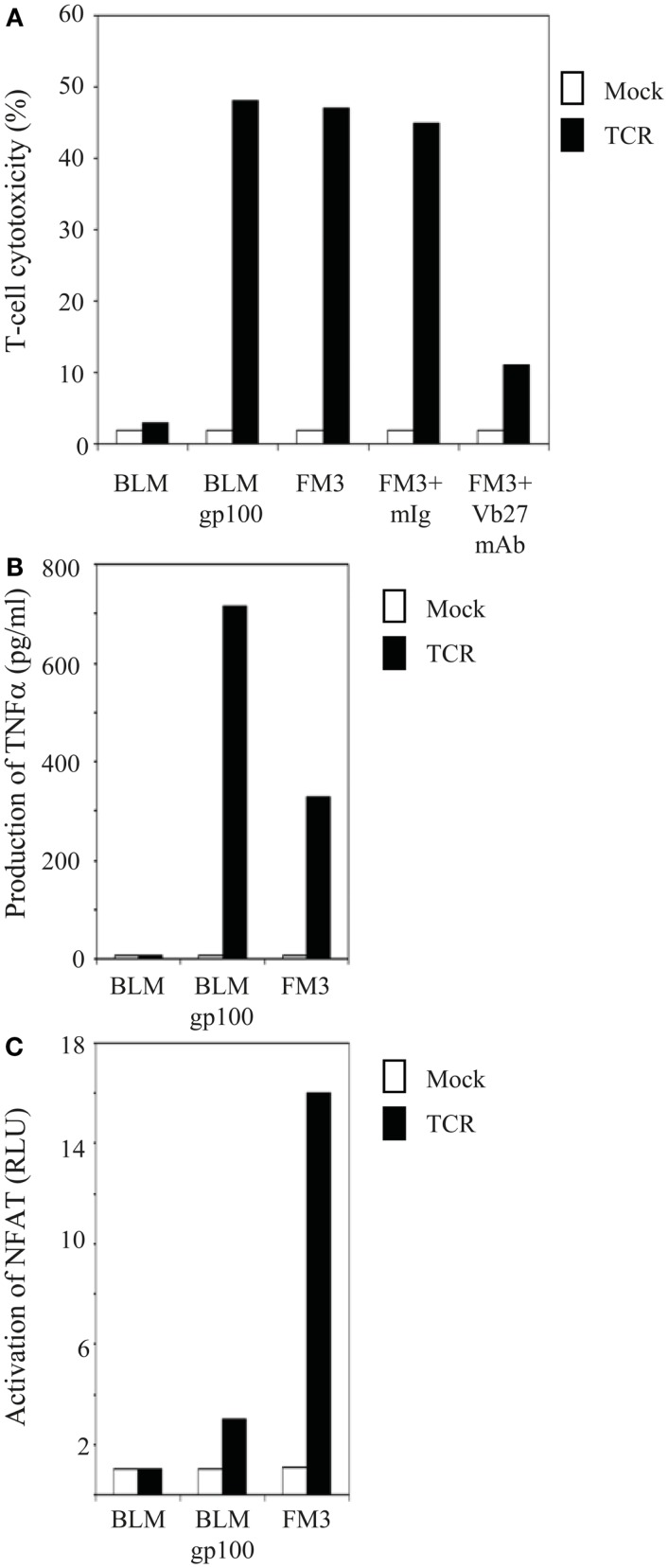
**T cells gene-engineered with a gp100/HLA-A2-specific TCR demonstrate antigen-specific reactivity against melanoma cells**. **(A)** TCR-transduced human T cells lyse gp100-positive, HLA-A2-positive melanoma cells. Primary human T cells transduced with TCR (black bars) or Mock genes (white bars) were tested in a 6 h ^51^Cr-release assay with the following target cells: the gp100-negative, HLA-A2-positive melanoma cell line BLM and the gp100-positive, HLA-A2-positive melanoma cell lines BLMgp100 and FM3. The effector to target cell ratio was 30:1. Inhibition studies were performed with T cells and FM3 target cells using anti-TCRVβ27 mAb (Vb27) or mIg. Specific lysis was calculated, and results of one (out of three) representative experiment are shown. **(B)** TCR-transduced human T cells produce TNFα in response to gp100-positive, HLA-A2-positive melanoma cells. T cells and melanoma cells as described in **(A)** were co-cultivated for 18 h at an effector to target cell ratio of 3:1, after which TNFα levels (in pg/ml) were determined in supernatants by ELISA. Results of one (out of three) representative experiment are shown. **(C)** TCR-transduced human T cells activate NFAT in response to gp100-positive, HLA-A2-positive melanoma cells. Jurkat T cells transduced with TCR (black bars) or Mock genes (white bars) were transfected with an NFAT reporter construct and subsequently co-cultivated for 6 h with the same target cells as described in **(A)** at an effector to target cell ratio of 2:1. Luciferase activities were determined in cell lysates and expressed relative to medium only [in Relative Light Units (RLU)]. Medium stimulations of TCR- and Mock-transduced Jurkat T cells were 0.024 and 0.018, respectively, and were both set to 1.0. Results of one (out of two) representative experiment are shown.

Reporter gene assays for NFAT were performed as described in detail elsewhere (35). In short, exponentially growing TCR/CD8-co-transduced Jurkat T cells (5 × 10^6^) were electroporated with a construct containing Firefly Luciferase coupled to six response elements of NFAT [FLuc-NFAT(RE)6]. Twenty hours post-transfection, Jurkat T cells were transferred to round-bottom 96-well plates at 2 × 10^5^ cells/well and co-incubated with target cells at 10^5^ cells per well for 6 h in RPMI 1640 medium supplemented with 1% BCS at 37°C and 5% CO_2_. FM3 cells were pre-incubated O/N with IFNγ (PeproTech, NJ, USA, 10 ng/ml) and IL-1β (PeproTech, 30 ng/ml), and co-cultivation of these melanoma cells with Jurkat T cells was performed in the presence of anti-CD28 mAb (clone 15E8, 2 μg/ml, Sanquin Blood Supply Foundation). As a positive control for activation of NFAT, T cells were stimulated with PMA and ionomycin. Following stimulation, cells were lysed and luciferase activities were determined. Luciferase activities were expressed relative to a non-stimulated condition (medium only, which was set to 1.0; Figure 1) or in absolute light units corrected for a non-stimulated condition (LU corrected for medium only; Figure 5).

### Presentation of the data

All assays, i.e., cytotoxicity, cytokine production, and NFAT activation assays have at least three data points of which mean values (with all values within 15% of mean) were used for graphical presentation. For each graph the experiment as a whole was repeated several times (as indicated) and data of a representative experiment was shown (the latter based on the mean value of triplicate data points per experiment).

## Results

### Single amino acid substitutions of the gp100 wt peptide do not affect T cell functions except for E3 to a substitution, which results in a null ligand

In order to study gp100 peptide requirements of various TCR-mediated responses, both primary human T cells and Jurkat T cells were retrovirally transduced with TCR α and β genes that originated from the gp100/HLA-A2-specific CTL clone 296, and MACsorted for high and equal levels of TCR expression. Flow cytometry with TCRVβ mAb showed that gp100 TCR expression levels were about 90% [mean fluorescence intensity (MFI): 103] and 93% (MFI: 214) for primary human T lymphocytes and Jurkat T cells, respectively (data not shown). CD8 expression on primary human T cells was>50% and Jurkat T cells were co-transduced with the human CD8α gene (expression level: 100%; MFI: 590) (data not shown). Antigen-specific responses of TCR-engineered T cells were validated at the level of T cell cytotoxicity, production of TNFα, and activation of NFAT. Figure 1A demonstrates that TCR-transduced primary human T lymphocytes were able to lyse gp100-positive, HLA-A2-positive but not gp100-negative, HLA-A2-positive melanoma cell lines. The antigen-specificity of this response was further confirmed by the use of anti-TCRVβ mAb (Figure 1A). In addition, Figures 1B,C demonstrate that TCR-transduced human T lymphocytes produced TNFα and activated the transcription factor NFAT in an antigen-specific manner. Mock-transduced T cells did not show cytotoxic reactivities, TNFα production, and NFAT activation in response to any of the tumor cell lines tested. Data shown in Figure 1 confirm previous data from our group (7, 35) and further validate the antigen-specificity and use of the CTL296-derived TCRαβ for our *in vitro* analyses of APLs.

**Figure 2:**
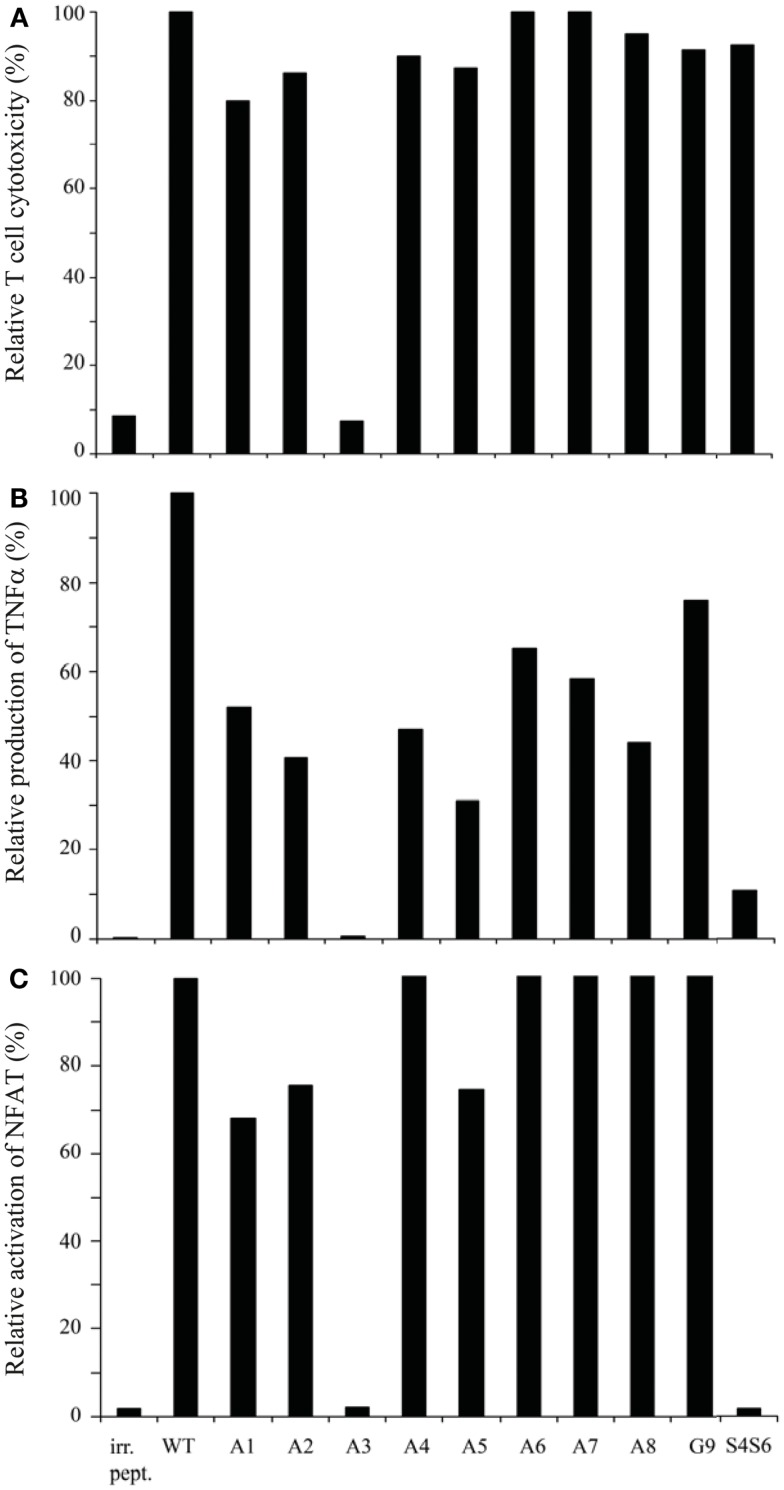
**gp100 APLs with single amino acid substitutions function as full agonists except for APL A3, which functions as a null ligand**. Human T cells transduced with gp100/HLA-A2 TCR genes were tested in **(A)** ^51^Cr-release, **(B)** TNFα production, and **(C)** NFAT reporter gene assays. Target cells used were T2 cells that were pre-incubated with 1 μM of gp100 wt peptide (wt), gp100 APLs A1–A8, and G9, or BMLF-1 wt peptide (irr. pept.). gp100 APLs are encoded as indicated in the Section “Materials and Methods.” In the ^51^Cr-release, TNFα production, and NFAT reporter gene assays, the effector to target cell ratios were 15:1, 3:1, and 2:1, respectively. T cell responses in all three assays are expressed relative to T2 target cells pulsed with gp100 wt peptide (specific lysis: 80%; production of TNFα: 1015 pg/ml; and activation of NFAT: 34.73 RLU, all set to 100%). Mock-transduced human T cells did not show activity in response to gp100 APLs (data not shown). Results of one (out of three) representative experiment are shown based on the mean value of triplicate data points. Note the null responses of the gp100 APL A3.

Using APLs with single amino acid substitutions, we studied the same three T cell responses with the following observations. *First*, all APLs (*n* = 9) sensitized T2 target cells for TCR-mediated lysis, except for APL with an E to A substitution at position 3 (i.e., APL A3) (Figure 2A). *Second*, although quantities of TNFα produced varied depending on the peptide used, all APLs, except for APL A3, induced production of this cytokine (Figure 2B). *Third*, again all APLs induced a clear activation of NFAT, except for APL A3 (Figure 2C). Mock-transduced T cells did not respond upon stimulation with any of the APLs tested, neither did TCR-transduced T cells respond to stimulation with BMLF-1 wt peptide (data not shown and Figure 2). Observations presented in Figure 2 extend earlier data showing that the cytotoxic responses of the parental CTL-296 versus gp100 APL with single amino acid mutations are preserved following TCR gene transfer into human T cells (7).

### A P4 and P6 to S substitution variant of the gp100 wt peptide functions as a partial agonist and dissects T cell cytotoxicity from production of cytokines and activation of NFAT

Next to the gp100 APLs with single amino acid substitutions, we generated an APL with a double amino acid mutation. The rationale behind designing this APL was to experimentally address whether replacement of both Proline amino acids, which are characterized by a rigid backbone, would affect and perhaps enhance T cell functions. Since Prolines at positions 4 and 6 in the wt gp100 peptide (YLEPGPVTA), when individually replaced by Alanine, did not alter function of T cells, we next replaced both Prolines by Serines, which are characterized by a more flexible backbone. This S4S6 peptide, when tested in the same set of assays as described above, was able to induce a cytotoxic T cell response and production of low levels of TNFα, but was not able to induce activation of NFAT in human T cells (Figures 2A–C).

**Figure 3:**
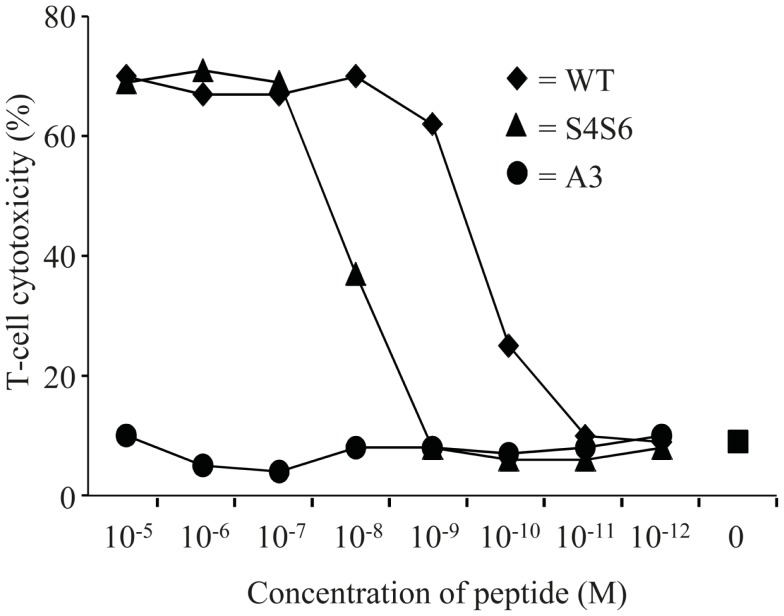
**The gp100 APL S4S6 is able to induce T cell cytotoxicity**. TCR-engineered T cells were tested in a ^51^Cr-release assay. T2 target cells were either non-loaded or loaded with titrated amounts of gp100 wt peptide (wt) or APLs A3 and S4S6 (range: 10^−5^–10^−12^ M peptide). T cell cytotoxicity was assayed after 6 h with an effector to target cell ratio of 30:1, after which specific lysis was calculated. Results of one (out of two) representative experiment are shown based on the mean value of triplicate data points.

**Figure 4:**
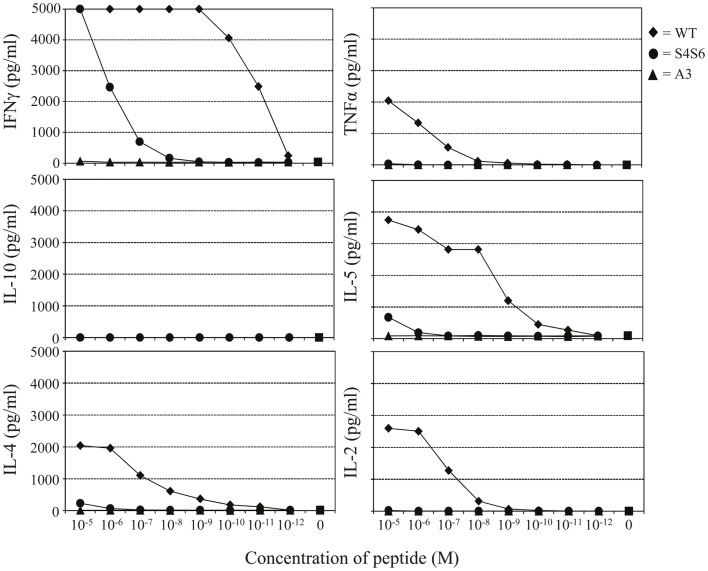
**The gp100 APL S4S6 induces a changed cytokine production profile**. TCR-engineered T cells were tested for the production of IFNγ, TNFα, IL-2, IL-4, IL-5, and IL-10 using a Cytokine Bead Array (CBA). T2 target cells were either non-loaded or loaded with titrated amounts of gp100 wt peptide (wt) or APLs A3 and S4S6 (range: 10^−5^–10^−12^ M peptide). Following an 18 h co-cultivation at an effector to target cell ratio of 3:1, supernatants were collected and measured for cytokine content. Results of one (out of three) representative experiment are shown based on the mean value of triplicate data points.

**Figure 5:**
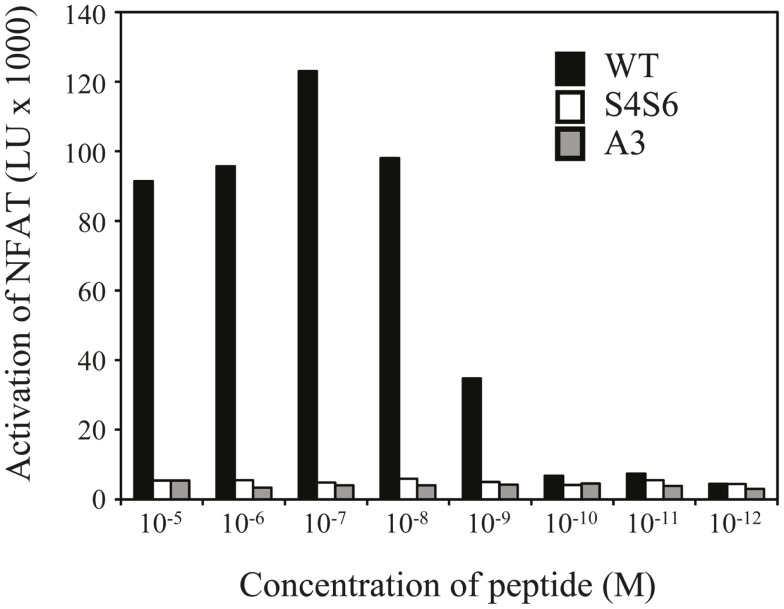
**The gp100 APL S4S6 is not able to induce activation of NFAT**. TCR-engineered Jurkat T cells were transfected with NFAT reporter and β-galactosidase constructs and subsequently co-cultured with T2 target cells that were either non-loaded or loaded with titrated amounts of gp100 wt peptide (wt) or APLs A3 and S4S6 (range: 10^−5^–10^−12^ M peptide). Following a 6 h co-cultivation at an effector to target cell ratio of 2:1, lysates were collected and measured for luciferase activities [depicted as light units (LU) corrected for medium only]. Results of one (out of two) representative experiment are shown based on the mean value of triplicate data points.

Findings with gp100 APLs suggest that APLs A1, A2, A4, A5, A6, A7, A8, G9 act as full agonists; APL A3 acts as a null ligand; and APL S4S6 acts as a partial agonist (Figure 2). To verify whether APLs A3 and S4S6 present a true null ligand and partial agonist, respectively, we performed extensive peptide dose-response studies. Results of repeated experiments were highly consistent and showed that APL A3 did not elicit T cell cytotoxicity (Figure 3), production of cytokines (Figure 4), and activation of NFAT (Figure 5) when tested over a 7-log range of peptide concentrations (10^−5^–10^−12^ M peptide). APL S4S6, however, induced decreased T cell cytotoxicity (Figure 3), an extremely lowered production of cytokines (Figure 4), but no activation of NFAT (Figure 5). When looking at wt peptide, we noted that over a large range of concentrations, T cell responses were comparable in all three assays, arguing that results with APLs are not due to differences in assay sensitivities. In example, wt peptide induced maximal T cell cytotoxicity, IFNγ production as well as NFAT activation over a 4-log range (10^−5^–10^−8^ M peptide), whereas these T cell responses became suboptimal from 10^−9^ to 10^−10^ M peptide onward, and were negligible from 10^−10^ to 10^−11^ M or lower. Instead, quantities of S4S6 peptide that were able to induce maximal T cell cytotoxicity were 10–100-fold higher when compared to wt peptide (Figure 3). With respect to cytokines, wt peptide selectively induced levels of IFNγ, and to a lesser extent TNFα, IL-2, IL-4, and IL-5, but not IL-10 (Figure 4). S4S6 peptide, however, triggered maximal production of IFNγ at concentrations that were 10^5^–10^6^-fold higher when compared to wt peptide (Figure 4). Production of IL-4 and IL-5 upon stimulation with S4S6 peptide was only detectable at the highest peptide concentrations used (at most 750 pg/ml when stimulating with 10^−5^ M peptide) (Figure 4). Using TNFα ELISAs, in our experience more sensitive than CBA, we again only observed detectable production of TNFα at the highest peptide concentrations used (at most 500 pg/ml when stimulating with 10^−5^ M peptide) (data not shown). Lastly, activation of NFAT was not observed for S4S6 peptide independent of the peptide concentration tested (Figure 5).

### APL S4S6-induced T cell functions show enhanced dependency on CD8

In an effort to explain our results with APLs A3 and S4S6, we examined their ability to bind to HLA-A2, their ability to bind (when complexed to HLA-A2) to the gp100 TCR, and to what extent T cell function, in case of S4S6, relies on the co-receptor CD8α.

**Figure 6:**
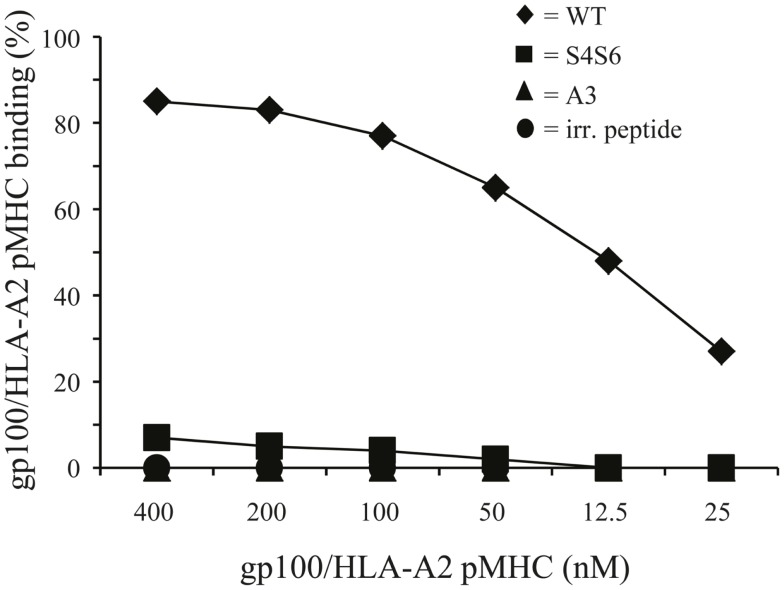
**Differential T cell responsiveness toward gp100 APLs is related to TCR’s ability to bind gp100/HLA-A2 complexes**. TCR-engineered T cells were stained with titrated amounts of PE-conjugated peptide-MHC multimers (range: 400–12,5 nM peptide-MHC complexes) and analyzed via flow cytometry. The gp100-HLA-A2 multimers comprise either gp100 wt peptide or APLs A3 and S4S6. The BMLF-1-HLA-A2 multimer served as a negative control. Binding of peptide-MHC multimers is analyzed as described in Section “Flow Cytometry and Cell-Sorting” and indicated in percentage. Percentages correspond to marker-positive T cells. Results of one (out of two) representative experiment are shown based on the mean value of triplicate data points.

**Figure 7:**
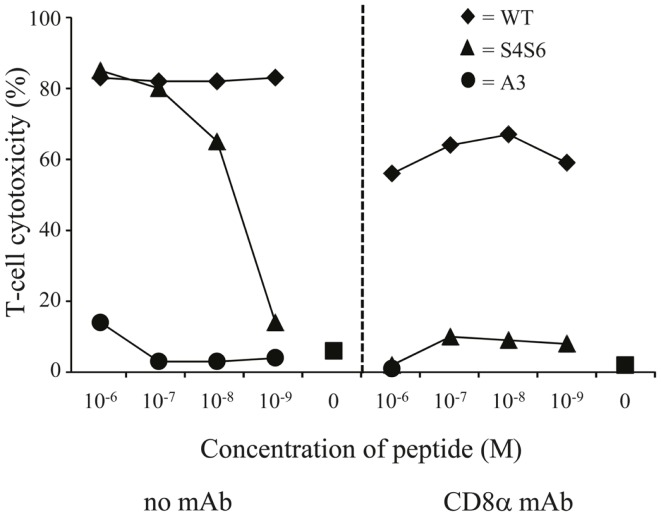
**The gp100 APL S4S6 renders the gp100/HLA-A2-specific TCR CD8 dependent**. TCR-engineered T cells were tested in a ^51^Cr-release assay as described in legend to Figure 3 either without or with blocking anti-CD8 mAb (left and right panels, respectively). T2 cells were either non-loaded or loaded with titrated amounts of gp100 wt peptide (wt) or APLs A3 and S4S6 (range: 10^−6^–10^−9^ M peptide). Results of one (out of two) representative experiment are shown based on the mean value of triplicate data points.

To study HLA-A2 binding of APLs, we performed an HLA-A2 stabilization assay using T2 cells. This assay showed that APLs A3 and S4S6 bind as efficient to HLA-A2 as does wt peptide (i.e., stabilization factors at 50 μM were 1.29, 1.14, and 1.25 for A3, S4S6, and wt peptide, respectively). The only APLs within our panel that bound significantly less to HLA-A2 are those that harbored a non-conservative mutation in one of the anchoring residues (i.e., APLs A2 and G9, but not V9, having stabilization factors at 50 μM of 0.00, 0.13, and 1.27, respectively). The abilities of APLs A3 and S4S6 to be stably bound by HLA-A2 allowed the synthesis of peptide-MHC multimers and detection of their binding by TCR-engineered T cells by flow cytometry. When analyzing the binding of titrated amounts of peptide-HLA-A2 multimers, we observed that neither A3-HLA-A2, nor (the negative control) BMLF-1-HLA-A2 were bound by T cells (Figure 6). In contrast, S4S6-HLA-A2 was bound by T cells, although to a lower extent than wt peptide-HLA-A2 (Figure 6). Finally, we established the contribution of CD8α to S4S6-induced T cell functions, to which end we have tested T cell cytotoxicity in the presence of a CD8α blocking antibody (Figure 7). Anti-CD8α antibody fully neutralized gp100 TCR-mediated cytotoxicity upon stimulation with S4S6 peptide, whereas decrease in cytolytic activity was negligible upon stimulation with wt peptide (Figure 7; right versus left panels).

## Discussion

In this study, we have characterized peptide requirements for T cell cytotoxicity, production of cytokines, and activation of NFAT. To this end, TCRαβ genes originating from the gp100_280–288_-HLA-A2-specific 296 CTL clone were transferred in human T cells, and receptor-mediated responses were tested versus gp100-positive and -negative melanoma cells (Figure 1) and a broad panel of gp100 APLs (Figure 2). We observed that APLs with single amino acid substitutions functioned as agonists. The E to A peptide mutant (i.e., APL A3) is an exception to this observation since it functions as a null ligand. The P to S double amino acid substitution variant (i.e., APL S4S6) functions as a partial agonist and is able to dissect cytotoxicity from cytokine production and NFAT activation.

Our observation that the performance of APLs with single amino acid substitutions was identical among the different T cell parameters tested, i.e., T cell cytotoxicity, production of cytokines, and activation of NFAT (Figure 2), was confirmed for a second gp100 TCR. Primary human T cells transduced with TCRαβ genes derived from the gp100_280–288_-HLA-A2-specific MPD CTL clone (37) showed cytolytic responses which were in complete accordance with TNFα production upon stimulation with APLs with single amino acid mutations [(7), and data not shown]. In case of the CTL-296-derived TCR, APL A3 behaved as a null agonist with respect to T cell cytotoxicity, production of cytokines, and activation of NFAT (Figure 2). This non-responsiveness is not caused by less efficient presentation of A3 peptide since the binding capacity of this mutant to HLA-A2 molecules is in the same range as that of the wt peptide. Rather, the non-responsiveness is caused by the TCR’s inability to bind A3-HLA-A2 complexes (Figure 6). The classification of APL A3 as a null agonist, and not as a potentially weak or partial agonist, is justified by the observation that peptide concentrations over a 7-log range did not affect T cell activities (Figures 3–5). In addition, we were unable to show an inhibitory effect of excess A3 peptide on wt peptide-induced responses in TCR-transduced human T cells, suggesting that this peptide variant is not acting as an antagonist (data not shown). The possibility that APL A3 acts as a supra-agonist, i.e., a null ligand that enhances the reactivation of memory CTL responses (38), is currently under investigation.

Altered peptide ligand S4S6 was classified as a partial agonist since it was able to induce cytotoxicity, production of only selected cytokines, but not activation of NFAT when compared to wt peptide (Figures 3–5). When using titrated amounts of peptide we demonstrated that 10–100-fold more S4S6 peptide was needed to induce cytotoxicity when compared to wt peptide (Figure 3). The decreased sensitivity of TCR-mediated cytotoxicity was relatively minor and at least a 1000-fold less when compared to the decreased sensitivities of TCR-mediated cytokine production and NFAT activation for S4S6 versus wt peptide (see below). Induction of cytotoxic activity is considered an early T cell response and generally requires small amounts of antigenic peptide and hardly any TCR down-regulation. Cytolytic proteins, e.g., perforin and granzymes, are pre-synthesized and thereby lower the threshold to trigger their release, which may explain the success of partial agonists to induce cytotoxicity (39–43). S4S6 peptide induced the production of IFNγ and to a lesser extent of IL-4, IL-5, and TNFα, but not IL-2 and IL-10 (Figure 4) or the activation of NFAT (Figure 5). In fact, TCR-mediated production of IFNγ required 10^5^–10^6^-fold higher concentrations of S4S6 when compared to wt peptide, and production of IL-4, IL-5, and TNFα required>10^6^-fold higher concentrations of S4S6, whereas production of IL-2, IL-10, and activation of NFAT could not even be measured at the highest concentration of S4S6 tested. These findings are in line with the notion that *de novo* synthesis of cytokines, which in many cases depends on NFAT activation, generally requires higher amounts of antigenic peptide and a much stronger TCR signal, i.e., high levels of TCR occupancy and TCR down-regulation (43). Production of cytokines and activation of NFAT, considered late T cell responses, may therefore not be triggered by a partial agonist. Partially agonistic peptides can selectively stimulate some T cell effector functions by inducing a pattern of signal transduction that is qualitatively different from the pattern induced by any concentration of the native peptide (9, 44–46). Partial agonistic signaling patterns are characterized by differential phosphorylation of TCR subunits, recruitment but no activation of ZAP-70, activation of MAP kinases (although for a shortened time period) and/or phenotypically distinct Ca^2+^ fluxes (11, 47, 48). A shortened period of MAP kinase activation and/or weakened Ca^2+^ flux could explain the observed lack of NFAT activation in T cells following stimulation with S4S6 peptide. Translocation of NFAT is reported to take place during “the TCR and co-receptor microclustering stage” in the formation of an immunological synapse (49). It would be interesting to find out whether S4S6 peptide would allow for TCR engagement but not proceeding to TCR microclustering and/or its coalescence into a central synapse. Another property of APL S4S6 may include its potential ability to induce T cell anergy. This state of T cell hyporesponsiveness is generally induced by triggering the TCR in the absence of sufficient T cell co-stimulation or in the presence of proficient T cell co-inhibition, and accompanied by the expression of anergy-associated genes, which subsequently contributes to impaired TCR signaling (50). Although there exist multiple forms of anergy, and it is mainly studied in CD4 T cells, the induction of anergy-associated genes appears to depend on the activation of NFAT. Hence, we argue that APL S4S6, because of its inability to activate NFAT, does not contribute to an anergic state of T cells.

The partial T cell responsiveness induced by APL S4S6 is not related to an altered ability of S4S6 peptide to bind to HLA-A2, but rather to a substantially decreased ability of the gp100 TCR to bind S4S6-HLA-A2 complexes (Figure 6). The new Serine residues, although not affecting binding to HLA-A2, apparently changed peptide-MHC conformation such that TCR chains showed a decreased fit for APL S4S6. Interestingly, APLs A2 and G9 with non-conservative amino acid substitutions at one of the peptide anchor positions to bind to HLA-A2 showed a significantly lowered stabilization of HLA-A2, yet were clearly able to induce cytotoxicity, TNFα production, and activation of NFAT (Figure 2). This apparent discrepancy most likely suggests that the CTL-296-derived TCR is of high affinity and that only a few peptide-loaded MHC class I molecules are needed to induce T cell activation. This would, in turn, further argue in favor of a dominant role for the interaction between peptide-MHC and TCR when compared to the interaction between peptide and MHC. Since the CD8α co-receptor can contribute to the stability of TCR:pMHC interactions and may, at least in part, compensate for a lowered affinity of a TCR for peptide-MHC (51, 52), we addressed whether S4S6 and wt peptide differed with respect to the CD8α-dependency of T cell cytotoxicity. In line with a decreased recognition by gp100 TCR, we observed that T cell responses toward APL S4S6 showed an enhanced involvement of CD8α (Figure 7). These observations extend those of Laugel and colleagues, who reported that T cell activation induced by strong, but not weak or partial agonists, does occur without CD8 co-activation (52). In fact, our results with APL S4S6 may suggest that the agonist potential of peptide mutants relates to the extent of binding by TCR and CD8α, a hypothesis that should be tested for multiple partial agonists.

Taken together, we have studied a novel panel of APLs in the context of TCR-engineered T cells and identified gp100_280–288_ APLs that act either as full agonists, a null ligand, or a partial agonist. In addition, our findings dissected T cell cytotoxicity from T cell cytokine production and NFAT activation and revealed that early T cell responses may require less peptide-MHC when compared to late T cell responses. Notably, our data suggest that partial agonists show a decreased binding by TCR and an enhanced dependency on CD8α, which may represent a novel mechanism behind the properties of partial agonists. Building on the current report, further design and testing of additional APLs may be necessary to advance the therapeutic application of APLs.

## Conflict of Interest Statement

The authors declare that the research was conducted in the absence of any commercial or financial relationships that could be construed as a potential conflict of interest.
